# Assessing the quality and completeness of reporting in health systems guidance for pandemics using the AGREE-HS tool

**DOI:** 10.7189/jogh.13.06050

**Published:** 2023-10-27

**Authors:** Luka Ursić, Marija F Žuljević, Miro Vuković, Nensi Bralić, Rea Roje, Jakov Matas, Antonija Mijatović, Damir Sapunar, Ana Marušić

**Affiliations:** 1 Department of Research in Biomedicine and Health, University of Split School of Medicine, Split, Croatia; 2 Center for evidence-based medicine, University of Split School of Medicine, Split, Croatia; 3 Department of Medical Humanities, University of Split School of Medicine, Split, Croatia; 4 Scientific Department, University Hospital of Split, Split, Croatia; 5 Department of Histology and Embryology, University of Split School of Medicine, Split, Croatia

## Abstract

**Background:**

During health emergencies, leading healthcare organisations, such as the World Health Organization (WHO), the European Centre for Disease Control and Prevention (ECDC), and the United States Centers for Disease Control and Prevention (CDC), provide guidance for public health response. Previous studies have evaluated clinical practice guidelines (CPGs) produced in response to epidemics or pandemics, yet few have focused on public health guidelines and recommendations. To address this gap, we assessed health systems guidance (HSG) produced by the WHO, the ECDC, and the CDC for the 2009 H1N1 and COVID-19 pandemics.

**Methods:**

We extracted HSG for the H1N1 and COVID-19 pandemics from the organisations’ dedicated repositories and websites. After screening the retrieved documents for eligibility, five assessors evaluated them using the Appraisal of Guidelines Research & Evaluation – Health Systems (AGREE-HS) tool to assess the completeness and transparency of reporting according to the five AGREE-HS domains: “Topic”, “Participants”, “Methods”, “Recommendations”, and “Implementability”.

**Results:**

Following the screening process, we included 108 HSG in the analysis. We observed statistically significant differences between the H1N1 and COVID-19 pandemics, with HSG issued during COVID-19 receiving higher AGREE-HS scores. The HSG produced by the CDC had significantly lower overall scores and single-domain scores compared to the WHO and ECDC. However, all HSG scored relatively low, under the median of 40 total points (range = 10-70), indicating incomplete reporting. The HSG produced by all three organisations received a median score <4 (range = 1-7) for the “Participants”, “Methods”, and “Implementability” domains.

**Conclusions:**

There is still significant progress to be made in the quality and completeness of reporting in HSG issued during pandemics, especially regarding methodological approaches and the composition of the guidance development team. Due to their significant impact and importance for healthcare systems globally, HSG issued during future healthcare crises should adhere to best reporting practices to increase uptake by stakeholders and ensure public trust in healthcare organisations.

During health emergencies, healthcare organisations, such as the World Health Organization (WHO), the European Centre for Disease Control and Prevention (ECDC), and United States Centers for Disease Control and Prevention (CDC), provide rapid clinical and public health guidelines [1-3]. In the case of the WHO, despite the expedited development process and the possible lack of evidence during emergencies, these guidelines are expected to adhere to transparent methods and evidence assessment approaches [1]. However, during the 2009 H1N1 pandemic, the WHO has been criticised by experts and the Council of Europe Parliamentary Assembly for lack of transparency regarding the names and conflicts of interest of WHO Emergency Committee Members who participated in guideline development processes [4,5]. The initial WHO guidance on mask-wearing during the coronavirus 2019 (COVID-19) pandemic was criticised as being confusing and self-contradictory [6]. More recently, the CDC leadership recognised their own errors in producing unclear guidance during the COVID-19 pandemic, especially due to a lack of transparent reporting of the accompanying scientific background [7]. In a recent qualitative study among public health experts in the European Union, participants observed that the ECDC produced unclear recommendations during the COVID-19 pandemic, but also acknowledged that its limited mandate prevented it from “providing strong standardized guidelines” for EU member states [3].

Previous studies have assessed clinical practice guidelines (CPGs) produced in response to public health emergencies. One study used the Appraisal of Guidelines for Research & Evaluation II (AGREE-II) tool to evaluate WHO emergency guidelines for four public health emergencies, including the H1N1 pandemic, and found they were of low overall quality and transparency, especially in reporting disclosures of interest, methodological processes, and applicability [8]. A recent assessment of COVID-19 CPGs developed by the WHO had similar findings [9]. Burda et al. [10] assessed CPGs approved by the WHO Guideline Review Committee and found that, despite frequent use of evidence reviews, specifics regarding the methodological steps (such as inclusion criteria and review approach) were often not reported.

Less attention has been given to the analysis of health systems guidance (HSG). Defined as “systematically developed statements produced at global or national levels to assist decisions about appropriate options for addressing a health systems challenge” [11], a HSG is any guideline, policy, recommendation, guidance, or similar document which “addresses a health systems challenge and provides recommendations or statements of action” in relation to the challenge. While developing the Appraisal of Guidelines for Research and Evaluation – Health Systems (AGREE-HS) instrument, Brouwers et al. [12] assessed 85 HSGs and found incomplete reporting on participants of the development process and methodology. The same tool was used to assess HSG on mental health and psychosocial support with similar findings, but this study also found the included HSG lack implementability [13]. Other studies looking at early COVID-19 pandemic infection and prevention control policies [14,15] have only provided a qualitative, summary overview of their content rather than a comprehensive analysis. To the best of our knowledge, there has been no systematic evaluation of the quality and reporting in HSG for pandemics as opposed to CPGs. To address this gap, we reviewed HSG produced by the WHO, the ECDC, and the CDC for the COVID-19 pandemic. We also analysed HSG issued by the same organisations for the 2009 H1N1 pandemic, to provide a historical perspective of HSG development.

## METHODS

### Search strategy

We searched the CDC Stacks repository, the WHO Institutional Repository for Information Sharing (IRIS), and the ECDC repository using a sensitive search strategy due to their low search capabilities, combining keywords such as “COVID-19” and “guidelines” through document full texts (Table S1 in the **Online Supplementary Document**). The end-point for the extraction was 17 March 2022. We also scraped the organisations’ websites dedicated to the H1N1 pandemic for documents not archived in the repositories using Python or R scripts (Table S1 and Text S1 in the **Online Supplementary Document**). For the CDC and the WHO, we conducted an additional search via PubMed limited to the Morbidity and mortality weekly report journal, which publishes CDC’s recommendations and reports, and the WHO Guidelines Approved by the Guidelines Review Committee. We exported the results into EndNote x9 (Clarivate, London, UK) or into Excel 2019 spreadsheets (Microsoft, Washington, USA) in the case of web scraped materials.

### Screening process

One researcher (LU) manually checked the documents exported in EndNote for duplicates, while those scraped from the websites were deduplicated using the *fromkeys* function in Python (Text S1 in the **Online Supplementary Document**). One researcher (LU) and a dedicated reviewer for each organisation (WHO – JM, ECDC – RR, CDC – MFŽ) screened the titles and abstracts of the retrieved documents, followed by their full texts. We resolved discrepancies through discussion, consulting a senior researcher (AM) for consensus. Three reviewers (LU, MFŽ, MV) then piloted the AGREE-HS tool on a sample of included documents (n = 15) to ensure consistency before conducting an additional eligibility screening. Any discrepancies were resolved through discussion.

### Inclusion/exclusion criteria

The inclusion and exclusion criteria were informed by the AGREE-HS tool [16] and focused on HSG for the H1N1 and COVID-19 pandemics for nonpharmacological interventions (e.g. masking, social/physical distancing, quarantine, movement restrictions, testing, tracing), infection prevention and control, healthcare system preparedness (including financing, management, etc.), implementation strategies for interventions (vaccine delivery, provision or delivery of healthcare, etc.), and resource allocation (vaccines, personal protective equipment, etc.) intended for policymakers within a healthcare system, healthcare providers, or healthcare managers.

We excluded documents related to the clinical management of COVID-19 or H1N1 (including vaccines, therapeutics, and other clinical interventions), methodology guidance for test sample collection and processing, technical specifications of testing devices, personal protective equipment, or ventilators, and tools or checklists which did not include guidelines, recommendations, or policies, but were related to public health measures. We also excluded guideline summaries, documents answering frequently asked questions, infographics, videos, or data sets on vaccination, infection, or death rates, risk assessments or risk reports, and guidance intended for public use.

### AGREE-HS evaluation

Five assessors (LU, MFŽ, MV, RR, NB) assessed the HSG using the AGREE-HS tool [17]. This tool had been validated and tested in several studies and applied in previous research on public health guidance [12,13,16,18]. The HSG were randomly assigned to the assessors, who received training on the tool and were provided articles on its development. Afterwards, they piloted the tool on five HSG prior to the full analysis, resolving issues or misunderstandings with the research team. The assessors were encouraged to leave a comment elaborating each grade for each domain. Each HSG was assessed by two reviewers. We used the final version of the HSG published by the end-point of the extraction (17 May 2022) in the analysis.

### Statistical analysis

We assessed and categorised the inter-rater agreement using Cohen’s kappa for two reviewers and Fleiss’ kappa for three or more reviewers, while also reporting percentage agreement, as suggested by previous literature [19,20] (Table S2 in the **Online Supplementary Document**). We reported descriptive statistics as medians and interquartile ranges for ordinal and continuous data and frequencies and percentages for categorical data. We presented median total AGREE-HS scores and calculated the transformed total scores following the formula “obtained score – minimum score/maximum score – minimum score” as per the AGREE-HS manual [17]. We compared the overall scores and the domain scores between the organisations using the Kruskal-Wallis test and conducted the Dwass-Steel-Critchlow-Fligner test for pairwise comparison. We compared the scores between the pandemics using Mann-Whitney’s U. We then used ordinal regression to determine which of the domains were predictors of an assessor recommending a HSG for use. We conducted the allocation and analyses in R, version 4.2.1 (R Core Team, Vienna, Austria), jamovi, version 2.3.16 (jamovi project, Sydney, Australia), and MedCalc, version 20.218 (MedCalc Software Ltd, Ostend, Belgium). We considered *P*-values <0.05 as statistically significant.

## RESULTS

### Screening process

**Figure 1:**
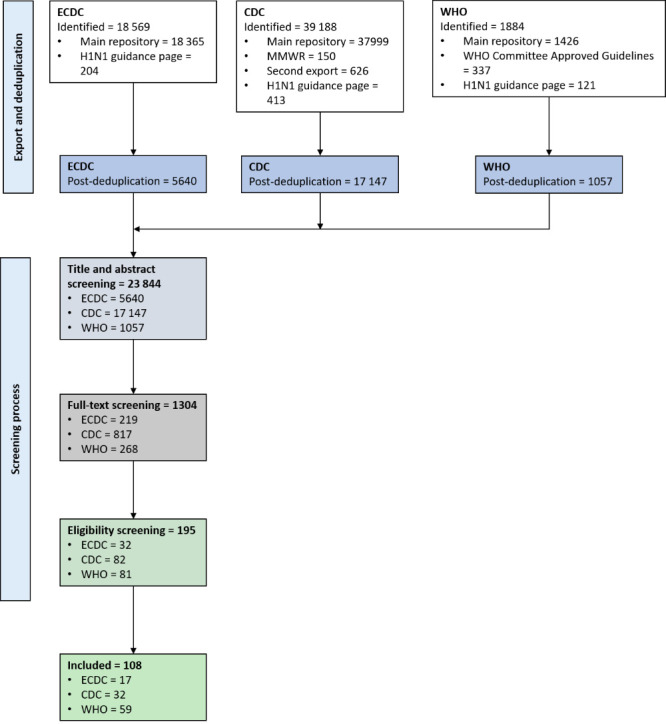
Study screening process. WHO – World Health Organization, ECDC – European Centre for Disease Control and Prevention, CDC – United States Centers for Disease Control and Prevention.

We exported 59 639 documents from all searched sources, with 23 844 remaining for the title/abstract screening after deduplication. After the initial screening, 1304 documents remained for full-text review; 195 documents were included for the pilot and the ensuing AGREE-HS eligibility screening. We included 108 HSG in the final analysis (**Figure 1** and Table S1 in the **Online Supplementary Document**). Fifty-nine were produced by the WHO, 32 by the CDC, and 17 by the ECDC. Among these, 16 were issued for the H1N1 and 92 for the COVID-19 pandemic (**Table 1**).

**Table 1:** Characteristics of the included health systems guidance

	Pandemic		Geographical scope	
**Organisation**	**H1N1**	**COVID-19**	**Global**	**Regional/national**
WHO	7	52	52	0
ECDC	2	15	0	17
CDC	7	25	2	30

CDC – United States Centers for Disease Control and Prevention, ECDC – European Centre for Disease Control and Prevention, WHO – World Health Organization

### AGREE-HS analysis

**Figure 2:**
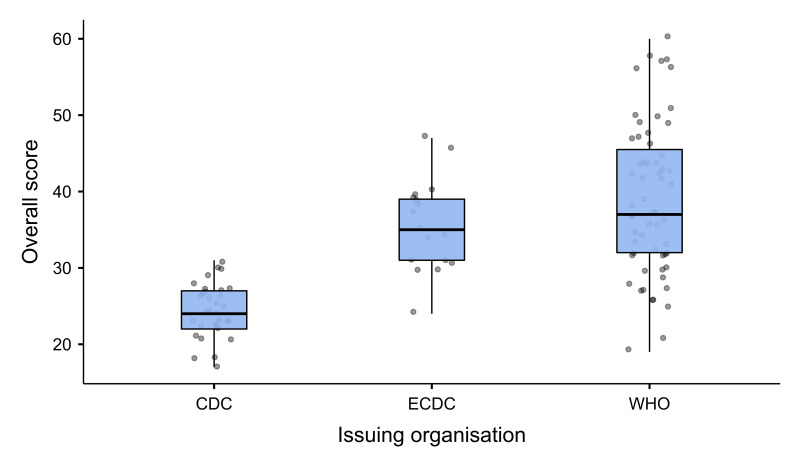
Box plot of overall AGREE-HS scores. Generated using jamovi, version 2.3.16 (jamovi project, Sydney, Australia). WHO – World Health Organization, ECDC – European Centre for Disease Control and Prevention, CDC – United States Centers for Disease Control and Prevention.

The HSG received low overall scores, under the mid-point of the total possible score (range = 10-70). For all organisation, the median scores were below 40, signifying incompleteness of reporting (**Table 2**). HSG produced by the CDC scored significantly lower compared to those produced by the WHO and ECDC, while there was no statistical difference between the overall scores for the ECDC and the WHO HSG. However, we observed significant dispersion in the scores for individual WHO HSG, suggesting that they were of varying quality (**Figure 2**). The difference in the overall scores between the pandemics was statistically significant, with HSG issued during the COVID-19 pandemic receiving higher scores.

**Table 2:** Overall AGREE-HS scores

			*P*-value			
	Raw total score, median (IQR)*	**Transformed total score, median (IQR)†**	**Overall**	**CDC vs ECDC**	**CDC vs WHO**	**ECDC vs WHO**
**Overall**	32.0 (26.0-42.0)	36.7 (26.7-53.3)				
**Organisation**			<0.001‡	<0.001‡	<0.001‡	0.455‡
CDC	24.0 (22.0-27.0)	23.3 (20.0-28.3)				
ECDC	35.0 (31.0-39.0)	41.7 (35.0-48.3)				
WHO	37.0 (32.0-45.5)	45.0 (36.7-59.2)				
**Pandemic**			0.015§	-	-	-
H1N1	27.0 (25.8-30.0)	28.3 (26.3-33.3)	-	-	-	-
COVID-19	33.5 (26.8-42.3)	39.2 (27.9-53.8)	-	-	-	-

AGREE-HS – Appraisal of Guidelines for Research and Evaluation – Health Systems, IQR – interquartile range, WHO – World Health Organization, ECDC – European Centre for Disease Control and Prevention, CDC – United States Centers for Disease Control and Prevention

*Median score for both assessors (range = 10-70), with 40 being considered as the mid-point.

†Calculated per the formula obtained score – minimum score/maximum score – minimum score as per the AGREE-HS manual [17].

‡Kruskal-Wallis test for overall and Dwass-Steel-Critchlow-Fligner test for pairwise comparison.

§Mann-Whitney’s U test.

**Figure 3:**
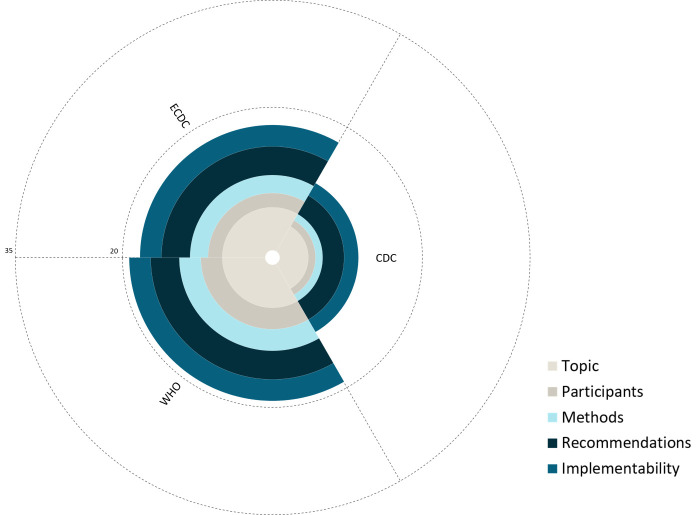
Coxcomb plot of specific AGREE-HS domain scores, stratified by organisation. The internal dashed line represents the midpoint of the total score, while the external line represents the maximum score. Generated using the Matplotlib package in Python, version 3.8.8. (Python software foundation, Delaware, USA). WHO – World Health Organization, ECDC – European Centre for Disease Control and Prevention, CDC – United States Centers for Disease Control and Prevention.

Regarding individual AGREE-HS domains, the ECDC and the WHO HSG received a median score above the mid-point (range = 1-7) only in the “Topic” domain, whereas the median score for the “Recommendation” domain was at the mid-point for both organisations. All organisations scored low in the “Implementability”, “Methods”, and “Participants” domains, indicating incomplete reporting. The CDC, which scored under the mid-point for all except the “Topic” domain, had statistically significant lower scores for all domains when compared to the ECDC and the WHO. We only observed a statistically significant difference between the ECDC and the WHO in the “Participants” domain, with the WHO HSG receiving higher scores (**Figure 3** and **Table 3**).

**Table 3:** Assessment and comparison of AGREE-HS domains

	AGREE-HS score, median (IQR)			*P*-value*			
	**CDC**	**ECDC**	**WHO**	**Overall**	**CDC vs ECDC**	**CDC vs WHO**	**ECDC vs WHO**
**Topic**	4.00 (3.00-6.00)	6.00 (4.25-6.00)	6.00 (5.00-7.00)	<0.001	<0.001	<0.001	0.801
**Participants**	1.00 (1.00-1.00)	2.00 (2.00-2.00)	3.00 (2.00-3.00)	<0.001	<0.001	<0.001	0.010
**Methods**	1.00 (1.00-2.00)	2.50 (2.00-3.00)	3.00 (2.00-4.00)	<0.001	<0.001	<0.001	0.534
**Recommendations**	3.00 (2.00-3.00)	4.00 (3.00-5.00)	4.00 (3.00-5.00)	<0.001	<0.001	<0.001	0.964
**Implementability**	2.00 (2.00-3.00)	3.00 (2.25-4.00)	3.00 (3.00-5.00)	<0.001	0.001	<0.001	0.587

AGREE-HS – Appraisal of Guidelines for Research and Evaluation – Health Systems, IQR – interquartile range, WHO – World Health Organization, ECDC – European Centre for Disease Control and Prevention, CDC – United States Centers for Disease Control and Prevention

*Kruskal-Wallis test for overall and Dwass-Steel-Critchlow-Fligner test for pairwise comparison.

Over half of the HSG (n = 56 (51.9%)) would not be recommended for use by either assessor, while 24 (22.2%) would be recommended for use either immediately (n = 4) or with modifications (n = 22) by both assessors. Of these 24, only four (3.7%) produced by the WHO were recommended unconditionally by both assessors, while the remaining 20 were either recommended unconditionally by one assessor and by the other after modification (ECDC: n = 4 (3.7%), WHO: n = 7 (6.5%)) or recommended following modifications by both assessors (WHO: n = 9 (8.3%)). None of the HSG produced by the CDC were recommended for use by both assessors, irrespective of modifications; only six would be recommended provided some modifications by at least one assessor, while the other assessor would still not recommend their use (**Table 4**).

**Table 4:** Recommendations for use*

		Organisation			Pandemic	
**Assessor recommendations**	**Overall**	**CDC**	**ECDC**	**WHO**	**COVID-19**	**H1N1**
Yes/yes	4 (3.7)	0 (0.0)	0 (0.0)	4 (3.7)	4 (3.7)	0 (0.0)
Yes/yes, with modifications	11 (10.2)	0 (0.0)	4 (3.7)	7 (6.5)	10 (9.3)	1 (0.9)
Yes, with modifications/Yes, with modifications	9 (8.3)	0 (0.0)	0 (0.0)	9 (8.3)	8 (7.4)	1 (0.9)
Yes/no	3 (2.8)	0 (0.0)	1 (0.9)	2 (1.9)	3 (2.8)	0 (0.0)
No/yes, with modifications	25 (23.1)	6 (5.6)	2 (1.9)	17 (15.7)	21 (19.4)	4 (3.7)
No/no	56 (51.9)	26 (24.1)	10 (9.3)	20 (18.5)	46 (42.6)	10 (9.3)

WHO – World Health Organization, ECDC – European Centre for Disease Control and Prevention, CDC – United States Centers for Disease Control and Prevention

*Reported as n (%) unless otherwise specified.

Based on the ordinal regression analysis, the “Participants” and “Methods” domains were predictors of assessors recommending a HSG for use (*P* < 0.001), with the “Methods” domain having the highest influence on the final recommendation. The “Implementability” domain showed only marginal statistical significance (*P* = 0.042) (**Table 5** and Tables S3-S4 in the **Online Supplementary Document**).

**Table 5:** Predictors of HSG being recommended for use by study assessors

Predictor/domain	Estimate (95% CI)	SE	Z	OR (95% CI)	*P*-value
Topic	0.260 (-0.1090, 0.655)	0.194	1.34	1.30 (0.897, 1.93)	0.180
Participants	0.539 (0.2344, 0.858)	0.158	3.41	1.71 (1.264, 2.36)	<0.001
Methods	1.064 (0.6707, 1.496)	0.210	5.08	2.90 (1.956, 4.46)	<0.001
Recommendations	0.245 (-0.1519, 0.639)	0.201	1.22	1.28 (0.859, 1.89)	0.223
Implementability	0.349 (0.0167, 0.692)	0.172	2.04	1.42 (1.017, 2.00)	0.042

OR – odds ratio, CI – confidence interval, SE – standard error

### Assessor’s comments

Due to the varying word length of the comments (n = 1176) from the AGREE-HS assessors, we did not conduct a qualitative analysis, but rather chose to select a few which reflected the assessor’s opinion of the HSG to provide a deeper insight on the specific aspects of the AGREE-HS domains they found lacking.

Regarding the “Participants” domain, the assessors highlighted that, while the ECDC and WHO mostly reported on the development team members’ names and institutions, they frequently failed to report on their conflicts of interest, their specific backgrounds/sectors, or the ways the influence of the funding organisation was managed:

“The development team members are mentioned alongside their institution, but with insufficient data on their backgrounds or sectors, which prohibits us from determining their stake or contribution to the development process. There is also no data on their conflicts of interest or the influence of the funding organization (or steps taken to limit it).”

However, the assessors observed that the CDC-produced HSG often did not report on the “Participants” domain at all, which could explain why it scored significantly lower in this domain than HSG produced by the WHO and ECDC:

“No information is given for this whatsoever, so this AGREE item is completely not addressed. No [information regarding] funder influence is mentioned, nor are any potential conflicts of interest.”

While the WHO and ECDC HSG scored higher than the CDC HSG in the “Methods” domain, the reporting was still incomplete; even in cases where literature reviews were conducted and where the evidence behind the recommendations was robust, the assessors observed that specific methods were usually not reported clearly:

“The methodological basis is a literature review process and a discussion with experts from relevant fields, alongside the implementation of existing guidelines with robust methodological processes. However, more details could have been provided regarding the review - for example, the screening process, search strategy, etc.”

“Despite a mention of a robust/transparent methodological approach, it is actually not presented very well; the evidence is composed by regular reviews and meetings of the development group, but we have no data on exact steps or consensus approaches. The evidence base, however, is robust and up-to-date, and linked to the recommendations, and shortcomings are discussed.”

Similarly to the “Participants” domain, the CDC HSG usually did not report on the methods at all, resulting in a significantly lower score:

“No methodological processes are presented in view of any review process for the evidence. There is also no mention of consensus in any form regarding the formulation of the recommendations (…) Evidence for the recommendations is not presented either, neither in the main guidance documents nor its annex.”

Even in cases when assessors found the “Recommendations” domain to be almost fully satisfied, the HSG did not clearly report on the plan to update the recommendations. Furthermore, the assessors observed that the outcomes of the recommendations were reported in a qualitative, general manner, with unclear thresholds and measurement parameters. They also noted that the societal impact of measures was not addressed, which could affect both the effectiveness of the recommendations and their acceptability in different contexts, and that plans for updating the HSG were not adequately presented:

“The recommendations are clear and succinct (…) annexes, accompanying guidance documents and prior versions contain enough information and clear-cut definitions (for example, concerning infection rates/levels of transmission in different settings) to make the guidance operationalizable. The only reason this guidance was not evaluated with the grade for highest quality is the plan for updating the recommendations, which is vague (i.e., only mentions that it will be updated when new knowledge emerges, without describing how or why whom).”

“Only qualitative descriptors are given regarding the specific outcomes expected from implementing the recommendations (…) no “end-point” is described with a specific threshold.”

“However, there is a lack of discussion on the impact of these measures on society as a whole, including the resources needed for implementing whole-of-society testing strategies, contact tracing, etc.”

In relation to the “Implementability” domain, the HSG generally did not discuss the affordability and cost-effectiveness of the measures, which was in turn related to their sustainability and their practical application in policy. The assessors suggested that specific cost projections could help make the recommendations more implementable;

“While barriers/enablers are discussed extensively, especially in view of limited evidence, there is a lack of discussion (at least an extensive one) about costs of interventions. This also affects transferability aspects, as low-income settings might not have substantial resource to dedicate to proper masking measures or public health masking.”

“The same is applicable to discussions regarding the sustainability of the travel-related measures; while it is mentioned and shortly discussed, such discussions warrant more detail. This could be done by projecting specific costs, giving thresholds/expected outcomes, and through similar means.”

## DISCUSSION

To the best of our knowledge, this is the first study to comprehensively assess the completeness and transparency of reporting in HSG issued by the WHO, CDC, and ECDC for the H1N1 and COVID-19 pandemics. We found that, despite more comprehensive reporting in the COVID-19 than the H1N1 pandemic, the HSG were lacking overall, especially in presenting the methodological process and disclosing information on the participants in HSG development, such as potential conflicts of interest.

Our findings are in line with a previous study on WHO emergency guidelines [8], which used the analogous AGREE II tool to assess 87 CPGs and found incomplete reporting on the development process and development team members’ conflicts of interest and independence from the funding organisation. A study of 18 CPGs, both national and international ones published by the WHO, had similar findings [9], as did a systematic review which encompassed the assessment of 626 CPGs [21], suggesting incomplete reporting. While limited in number due to the relative novelty of the AGREE-HS tool, studies assessing HSG on mental health and psychosocial support [13] and HSG produced by the WHO, NICE, and other organisations for varying public health challenges [12,22] also had similar findings. These shortcomings could significantly influence guideline uptake, as Kastner et al. [23] report that “Stakeholder involvement” (encompassing clear reporting of conflicts of interest and funding) and “Evidence synthesis” (encompassing clear reporting of methods) are two of six key domains influencing the uptake of CPGs. This is in line with our finding that the “Methods” and “Participants” domains were significant predictors of a HSG being recommended for use by our study assessors. However, a systematic review on the use of the AGREE II tool for CPGs also found that the “Rigour of Development” domain (analogous to the “Methods” domain in the AGREE-HS tool) predicted recommendations for use, but not the “Editorial Independence” domain (analogous to the “Participants” domain in our study) [24], which differs from our findings.

We also found overall low scores for the “Implementability” of the recommendations in the HSG due to a lack of considerations for cost-effectiveness analyses and sustainability considerations. Additionally, this domain was a marginally significant predictor of a HSG being recommended for use. In their systematic reviews of CPG assessments using the AGREE II tool, Alonso-Coello et al. [21] found low scores on the “Applicability” domain (analogous to the “Implementability” domain in our study), while Hoffmann-Eßer et al. [24] found it to be a statistically significant predictor of a HSG being recommended for use. Studies using the AGREE-HS tool on HSG similarly found low scores on the “Implementability” domain [12,13]. According to a recent scoping review, a clear implementation plan with actionable steps is necessary for policymakers to implement evidence-based guidance into policy [25]. In developing future HSG, healthcare organisations should consider how policymakers adapt and use guidelines, systematic reviews, and evidence in general while developing healthcare policies [26-28] and adapt their HSG accordingly.

Despite the emergent nature of public health crises, guidelines produced in such contexts are expected to adhere to rigorous development processes [29]. According to the WHO Handbook for Guideline Development, emergency (rapid response), rapid advice, and interim guidelines must all adhere to standard WHO guideline development processes, which include robust systematic reviews, guideline development group meetings and assessments, and clear reporting of the development group members’ conflicts of interest, among other methodological steps [30]. Although CDC interim guidelines are not necessarily meant to adhere to their standard development processes, which include rigorous systematic reviews, evidence evaluations, and processes for evaluating conflicts of interest, their developers are encouraged to apply and adapt them nonetheless [31]. Although we are not aware of standardised development processes within the ECDC, they should likely be analogous to those used at the WHO and the CDC. Our findings do not indicate that these processes were not adhered to, but rather that they were not transparently or fully reported. For example, the HSG issued by the CDC was often difficult to navigate and not accompanied by a scientific rationale or evidence, as confirmed by CDC’s recent internal revision [7], which led to low scores on the “Methods” domain in our study. During future crises, healthcare organisations and HSG developers should adhere to best practices in reporting and transparency, such as the ones outlined in the WHO Handbook for Guideline Development [30], irrespective of possible limitations in evidence or the incomplete use of standard guideline development methodologies due to the need for expedited HSG during public health crises. Lessons from the H1N1 and COVID-19 pandemics [5,7,32] on the need for transparency and rigorous reporting should inform future health crises responses to ensure public trust and improve uptake of evidence-based guidelines among policymakers, as suggested by the zero draft of the upcoming WHO pandemic treaty [33].

### Strengths and limitations

This study has several strengths and limitations. Regarding the strengths, we conducted a comprehensive, exhaustive search of multiple repositories, websites, and official publications of the WHO, CDC, and ECDC using a sensitive search strategy, with two reviewers screening all extracted guidance to ensure the inclusion of all relevant documents. We also conducted an additional eligibility screening following the pilot of the AGREE-HS tool, to ensure its uniform applicability on all HSG. The HSG were also randomised among the assessors, after which each was assessed independently by two assessors who were previously trained in the use of the AGREE-HS tool and who piloted it prior to the full analysis. We also looked at the HSG issued by three highly relevant organisations published for two pandemics, thus offering both a contemporary and historical perspective on the completeness and transparency of reporting in HSG for pandemics.

Despite our comprehensive, sensitive search strategy, and the inclusion of several sources, it is possible we overlooked certain HSG for inclusion due to the limited search capabilities of the repositories, possible omissions of archiving and indexation, and other shortcomings. Moreover, we found fewer HSG for the H1N1 than the COVID-19 pandemic, which could be attributed to fewer HSG being produced or archived in the repositories. Regarding the analysis itself, as is the case with any AGREE tools for assessing CPGs or HSG [34], the assessment process is subjective and depends on the number of assessors, their background, expectations, and other factors, so our results should be interpreted with caution. We also used two assessors in the process; while this is on the lower end of the recommended number of assessors (range = 2-4) [17], the AGREE development team suggested that it should be sufficient to properly evaluate the domains for the AGREE tools in general [34]. Notably, our analysis does not indicate that, for example, proper methodological steps were not taken or that conflicts of interests of the development team were not checked; it merely indicates an incompleteness of reporting that should be addressed in the future. Our analysis also accounts only for HSG published by 17 May 2022, so it does not include any published during the later stage of the COVID-19 pandemic, when more evidence was available. Furthermore, all HSG published during both pandemics is interim, thus subject to a lack of evidence and pressure on the issuing organisations to provide recommendations during crises, which possibly explains the low score on the “Methods” domain for all HSG. However, previous research has suggested that the crisis context should not affect the transparency and rigour in reporting CPGs or any guidelines in general, as clear reporting of methodologies is possible, even when the methods themselves might not be rigorous [1,8].

## CONCLUSIONS

We found incomplete reporting in the HSG produced by the WHO, ECDC, and CDC for the H1N1 and COVID-19 pandemics, especially in view of the methodologies used to develop the HSG, the participants of the development process and their conflicts of interest, the role of the funder, and the overall implementability of the guidance. During future healthcare crises, these organisations should implement better reporting practices to improve transparency, increase guideline uptake, and ensure public trust.

## Additional material


Online Supplementary Document
