# Supplementary material for: Assessing the quality and completeness of reporting in health systems guidance for pandemics using the AGREE-HS tool
Source: J Glob Health. 2023 Oct 27;13:06050. doi: 10.7189/jogh.13.06050 (PMC10602204; doi:10.7189/jogh.13.06050)
Supplement: Online Supplementary Document [file jogh-13-06050-s001.zip › jogh-13-06050-s001-OSD.pdf]

Table S1. Search strategy and retrieved documents

Keywords/search string	Modifiers	Date of search	Time span*	Results
**CDC**				
CDC Stacks (https://stacks.cdc.gov/)				
pandemic AND guideline	Document Full Text	29. 11. 2021.		657
pandemic AND guidelines	Document Full Text	29. 11. 2021.		2922
pandemics AND guideline	Document Full Text	29. 11. 2021.		152
pandemics AND guidelines	Document Full Text	29. 11. 2021.		539
pandemic AND recommendation	Document Full Text	29. 11. 2021.		1508
pandemic AND recommendations	Document Full Text	30. 11. 2021.		4089
pandemics AND recommendation	Document Full Text	30. 11. 2021.		288
pandemics AND recommendations	Document Full Text	30. 11. 2021.		652
pandemic AND policy	Document Full Text	30. 11. 2021.		3023
pandemic AND policies	Document Full Text	30. 11. 2021.		3149
pandemics AND policy	Document Full Text	30. 11. 2021.		584
pandemics AND policies	Document Full Text	30. 11. 2021.		458
H1N1 AND guideline	Document Full Text	1. 12. 2021.		380
H1N1 AND guidelines	Document Full Text	1. 12. 2021.		1530
H1N1 AND recommendation	Document Full Text	1. 12. 2021.		971
H1N1 AND recommendations	Document Full Text	1. 12. 2021.		2335
H1N1 AND policy	Document Full Text	1. 12. 2021.		1452
H1N1 AND policies	Document Full Text	1. 12. 2021.		1007
COVID-19 AND guideline	Document Full Text	1. 12. 2021.		206
COVID-19 AND guidelines	Document Full Text	1. 12. 2021.		1426
COVID-19 AND recommendation	Document Full Text	1. 12. 2021.		669
COVID-19 AND recommendations	Document Full Text	1. 12. 2021.		2721
COVID-19 AND policy	Document Full Text	1. 12. 2021.		1486
COVID-19 AND policies	Document Full Text	2. 12. 2021.		5795
Total				37999
Additional search to 17. 03. 2022.				

pandemic AND guideline	Document Full Text	25. 10. 2022.	29. 11. 2021. to 17. 03. 2022	6
pandemic AND guidelines	Document Full Text	25. 10. 2022.	29. 11. 2021. to 17. 03. 2022	25
pandemics AND guideline	Document Full Text	25. 10. 2022.	29. 11. 2021. to 17. 03. 2022	1
pandemics AND guidelines	Document Full Text	25. 10. 2022.	29. 11. 2021. to 17. 03. 2022	9
pandemic AND recommendation	Document Full Text	25. 10. 2022.	29. 11. 2021. to 17. 03. 2022	19
pandemic AND recommendations	Document Full Text	25. 10. 2022.	30. 11. 2021. to 17. 03. 2022	51
pandemics AND recommendation	Document Full Text	25. 10. 2022.	30. 11. 2021. to 17. 03. 2022	12
pandemics AND recommendations	Document Full Text	25. 10. 2022.	30. 11. 2021. to 17. 03. 2022	50
pandemic AND policy	Document Full Text	25. 10. 2022.	30. 11. 2021. to 17. 03. 2022	40
pandemic AND policies	Document Full Text	25. 10. 2022.	30. 11. 2021. to 17. 03. 2022	33
pandemics AND policy	Document Full Text	25. 10. 2022.	30. 11. 2021. to 17. 03. 2022	22
pandemics AND policies	Document Full Text	25. 10. 2022.	30. 11. 2021. to 17. 03. 2022	16
H1N1 AND guideline	Document Full Text	25. 10. 2022.	1. 12. 2021. to 17. 03. 2022	2
H1N1 AND guidelines	Document Full Text	25. 10. 2022.	1. 12. 2021. to 17. 03. 2022	4
H1N1 AND recommendation	Document Full Text	25. 10. 2022.	1. 12. 2021. to 17. 03. 2022	4
H1N1 AND recommendations	Document Full Text	25. 10. 2022.	1. 12. 2021. to 17. 03. 2022	6

H1N1 AND policy	Document Full Text	25. 10. 2022.	1. 12. 2021. to 17. 03. 2022	8
H1N1 AND policies	Document Full Text	25. 10. 2022.	1. 12. 2021. to 17. 03. 2022	4
COVID-19 AND guideline	Document Full Text	25. 10. 2022.	1. 12. 2021. to 17. 03. 2022	8
COVID-19 AND guidelines	Document Full Text	25. 10. 2022.	1. 12. 2021. to 17. 03. 2022	39
COVID-19 AND recommendation	Document Full Text	25. 10. 2022.	1. 12. 2021. to 17. 03. 2022	38
COVID-19 AND recommendations	Document Full Text	25. 10. 2022.	1. 12. 2021. to 17. 03. 2022	134
COVID-19 AND policy	Document Full Text	25. 10. 2022.	1. 12. 2021. to 17. 03. 2022	63
COVID-19 AND policies	Document Full Text	25. 10. 2022.	1. 12. 2021. to 17. 03. 2022	32
Total				626
H1N1 page (https://www.cdc.gov/h1n1flu/)	N/A			413
MMWR (via PubMed)	N/A			150
**TOTAL**				39118
**ECDC**				
ECDC repository (https://www.ecdc.europa.eu/en/search?s=)				
COVID-19	Data, News, Publications	08. 04. 2022.		911
COVID-19 recommendations	Data, News, Publications	08. 04. 2022.		2150
COVID-19 guidelines	Data, News, Publications	08. 04. 2022.		1507
H1N1	Data, News, Publications	08. 04. 2022.		660
H1N1 recommendations	Data, News, Publications	08. 04. 2022.		2262

H1N1 guidelines	Data, News, Publications	08. 04. 2022.	1328
pandemic guidelines	Data, News, Publications	08. 04. 2022.	1354
pandemic recommendations	Data, News, Publications	08. 04. 2022.	2284
Topic filter search (*Severe acute respiratory syndrome (SARS), SARS-CoV-2, SARS-CoV-2 variants, COVID-19, Coronavirus, Influenza A(H1N1), Influenza A(H1N1) 2009, Swine origin influenza, Influenza in humans, Swine origin, Influenza in humans, pandemic*)	Data, News, Publications	15. 04. 2022.	5909
H1N1 webpage (https://www.ecdc.europa.eu/en/seasonal-influenza/2009-influenza-h1n1)	N/A		204
**TOTAL**			18569
**WHO**			
WHO IRIS (https://apps.who.int/iris/)			
Title: recommendations CONTAINS Subject: COVID-19	None/All of IRIS	17. 03. 2022.	86
Title: recommendation CONTAINS Subject: COVID-19	None/All of IRIS	17. 03. 2022.	86
Title: guidelines CONTAINS Subject: COVID-19	None/All of IRIS	17. 03. 2022.	15
Title: guideline CONTAINS Subject: COVID-19	None/All of IRIS	17. 03. 2022.	15
Title: recommendations CONTAINS Subject: H1N1	None/All of IRIS	17. 03. 2022.	1
Title: recommendation CONTAINS Subject: H1N1	None/All of IRIS	17. 03. 2022.	1
Title: guidelines CONTAINS Subject: H1N1	None/All of IRIS	17. 03. 2022.	2
Title: guideline CONTAINS Subject: H1N!	None/All of IRIS	17. 03. 2022.	2
Subject: recommendations CONTAINS Subject: COVID-19	None/All of IRIS	17. 03. 2022.	0
Subject: recommendation CONTAINS Subject: COVID-19	None/All of IRIS	17. 03. 2022.	0
Subject: guidelines CONTAINS Subject: COVID-19	None/All of IRIS	17. 03. 2022.	538
Subject: guideline CONTAINS Subject: COVID-19	None/All of IRIS	17. 03. 2022.	538
Subject: recommendations CONTAINS Subject: H1N1	None/All of IRIS	17. 03. 2022.	0
Subject: recommendation CONTAINS Subject: H1N1	None/All of IRIS	17. 03. 2022.	0
Subject: guidelines CONTAINS Subject: H1N1	None/All of IRIS	17. 03. 2022.	6

Subject: guideline CONTAINS Subject: H1N1	None/All of IRIS	17. 03. 2022.	6
Title: recommendations CONTAINS Subject: Pandemic	None/All of IRIS	17. 03. 2022.	3
Title: recommendation CONTAINS Subject: Pandemic	None/All of IRIS	17. 03. 2022.	3
Title: guidelines CONTAINS Subject: Pandemic	None/All of IRIS	17. 03. 2022.	2
Title: guideline CONTAINS Subject: Pandemic	None/All of IRIS	17. 03. 2022.	2
Subject: recommendations CONTAINS subject: pandemic	None/All of IRIS	17. 03. 2022.	0
Subject: recommendation CONTAINS subject: pandemic	None/All of IRIS	17. 03. 2022.	0
Subject: guidelines CONTAINS subject: pandemic	None/All of IRIS	17. 03. 2022.	60
Subject: guideline CONTAINS subject: pandemic	None/All of IRIS	17. 03. 2022.	60
H1N1 webpage (https://www.who.int/emergencies/situations/influenza-a-(h1n1)-outbreak)	N/A		121
WHO Committee Approved Guidelines (https://www.ncbi.nlm.nih.gov/books/NBK132015/)	N/A		337
**TOTAL**			1884

CDC – United States Centers for Disease Control and Prevention, ECDC – European Centres for Disease Control and Prevention, WHO – World Health Organization, MMWR – MMWR. Morbidity and Mortality Weekly Report, N/A – not applicable

*No limitation set to time span unless otherwise indicated.

Table S2. Inter-rater agreement

	**Total**	**Included**	**Excluded**	**Included**	**Excluded**	**Included**	**Excluded**	**IRR (% agreement)**	**Strength of agreement**
**ECDC***		**LU**	**LU**	**RR**	**RR**				
Title/abstract screening	5640	201	5439	191	5449			0.87 (99.10)	Strong
Full-text screening	219	54	165	55	164			0.81 (93.20)	Strong
**WHO***		**LU**	**LU**	**JM**	**JM**				
Title/abstract screening	1057	266	791	277	780			0.93 (97.60)	Almost Perfect
Full-text screening	268	142	126	154	114			0.86 (93.30)	Strong
**CDC***		**LU**	**LU**	**MFŽ**	**MFŽ**				

Title/abstract screening	17147	780	16367	791	16356			0.98 (99.90)	Almost Perfect
Full-text screening	817	189	628	171	646			0.70 (90)	Moderate
**AGREE pilot†**		**LU**	**LU**	**MFŽ**	**MFŽ**	**MV**	**MV**		
	195	111	84	106	89	115	80	0.70 (78.50)	Substantial

IRR – inter-rater agreement

*Cohen's kappa. Strength of agreement categorised following McHugh's interpretation [1].

†Fleiss' kappa for multiple raters. Strength of agreement categorised following Landis and Koch's interpretation [2].

Table S3. Model fit for ordinal regression

				**Overall model test**		
**Model**	**Deviance**	**AIC**	**McFadden's R^2**	**χ^2**	**df**	***P*-value**
1	195	209	0.479	179	5	<0.001

df – degrees of freedom

Table S4. Overall results for the five AGREE-HS domains

	**AGREE-HS score, median (95% CI)**
**Topic**	6.00 (5.00-6.00)
**Participants**	2.00 (1.00-2.00)
**Methods**	2.00 (2.00-3.00)
**Recommendations**	3.00 (3.00-4.00)
**Implementability**	3.00 (3.00-3.00)

CI – confidence interval

Text S1. Explanation of Python and R scripts

Python

Using ECDC's search engine (https://www.ecdc.europa.eu/en), we performed 18 queries applying the following keywords: "COVID-19", "COVID-19 recommendations", "COVID-19 guidelines", "H1N1", "H1N1 recommendations", "H1N1 guidelines", "pandemic recommendations", and "pandemic guidelines", and the following filters: "Severe acute respiratory syndrome (SARS)", "SARS-CoV-2", and "SARS-CoV-2 variants", "COVID-19", "Coronavirus", "Influenza A (H1N1)", "Influenza A (H1N1)2009", "Swine-origin influenza", "Influenza in Humans, Swine Origin", and "Influenza in humans, pandemic".

For each query we used its default URL address and concatenated the address with page numbers. For example, for "COVID-19 recommendations", the default 'https://www.ecdc.europa.eu/en/search?search_section%5B1382%5D=1382&search_section%5B1307%5D=1307&search_section%5B1244%5D=1244&s=COVID-19%20recommendations&page=' address was concatenated with numbers ranging from 0 to 217, as 216 was the number of pages for "COVID-19 recommendations" query in the moment when the queries were performed. After generating query website URLs, we found 10 sections on each website. These sections represented information on articles such as the article's title, short abstract and hyperlink. In order to retrieve the hyperlinks, we scraped the obtained URL pages using requests (https://docs.python-requests.org/en/latest/) and Beautiful Soup (https://beautiful-soup-4.readthedocs.io/en/latest/) HTTP libraries for Python. We used the *fromkeys* function in Python (https://python-reference.readthedocs.io/en/latest/docs/dict/fromkeys.html) to deduplicate the results. Articles' titles and hyperlinks were saved in a tabular pandas.DataFrame data structure (https://pandas.pydata.org/docs/reference/api/pandas.DataFrame.html) and exported to a .xlsx file.

The process of querying was done automatically on the 15 May 2022, for which we wrote a Python script in Python, version 3.8.8 (Python software foundation, Delaware, USA) (https://www.python.org/downloads/release/python-388/). A total of 18 363 article was retrieved; 5445 were left after deduplication across searches.

R

We used R, version 4.2.1 (R Core Team, Auckland, New Zealand) to scrape the "Publications" section of the H1N1 dedicated website (https://www.ecdc.europa.eu/en/seasonal-influenza/2009-influenza-h1n1) using the *RSelenium* and *tidyverse* packages. We then selected the "Publications" subsection and iterated over the "Load More" button to reveal all possible articles, after which we scraped their titles and hyperlinks into a tabular format within R, which we then exported as a .csv. Through this method, we retrieved a total of 204 articles; 195 remained following deduplication, amounting to a total of 5640 articles.

REFERENCES

1. McHugh ML. Interrater reliability: the kappa statistic. Biochem Med (Zagreb). 2012;22(3):276-82.
2. Landis JR, Koch GG. The measurement of observer agreement for categorical data. Biometrics. 1977 Mar;33(1):159-74.
